# Work-related stress among nurses in the COVID-19 pandemic: What are the contributing factors?

**DOI:** 10.1590/0034-7167-2022-0586

**Published:** 2023-12-04

**Authors:** Tita Rohita, Nursalam Nursalam, Muhammad Hadi, Iqbal Pramukti, Dedeng Nurkholik, Arifah Septiane, Idyatul Hasanah, Ika Adelia Susanti

**Affiliations:** I Universitas Airlangga, Faculty of Nursing. Surabaya, Indonesia; II Universitas Galuh, Faculty of Health Sciences. Ciamis, Indonesia; III Universitas Muhammadiyah Jakarta, Faculty of Nursing. Jakarta, Indonesia; IV Universitas Padjadjaran, Faculty of Nursing, Department of Community Health Nursing. Sumedang, Indonesia; V Mataram Institute of Health Science, Department of Nursing. Mataram, Indonesia; VI Universitas Dr. Soebandi, Faculty of Health Science. Jember, Indonesia

**Keywords:** COVID-19, Nurses, Occupational Stress, Stress, Workload, COVID-19, Enfermeiras, Estresse Ocupacional, Estresse, Carga de Trabalho, COVID-19, Enfermeras, Estrés Laboral, Estrés, Carga de Trabajo

## Abstract

**Objective:**

To analyze the contributing factors of work-related stress among nurses in the COVID-19 pandemic.

**Methods:**

A cross-sectional study was conducted with 101 nurse practitioners from two hospitals in West Java, Indonesia. We distributed an online questionnaire to evaluate work-related stress and the data were analyzed using ordinal logistic regression.

**Results:**

More than half of the nurses experienced moderate work-related stress. The study revealed that nurses aged over 35 years old had a lower likelihood of experiencing work-related stress (AOR: 0.173; 95%CI: 0.038-0.782). Married nurses had a higher likelihood (AOR: 7.156; 95% CI: 1.456-35.163). Additionally, nurses with low and moderate workloads had a lower likelihood (AOR: 0.003; 95%CI: 0.000-0.051) and (AOR: 0.025; 95%CI: 0.005-0.116), respectively.

**Conclusion:**

The consideration of age, marital status, and workload is essential in effectively addressing work-related stress among nurse practitioners.

## RESUMO

**Objetivo:**

Analisar os fatores contribuintes do estresse laboral entre enfermeiros na pandemia de COVID-19.

**Método:**

Estudo transversal conduzido com 101 enfermeiros de dois hospitais em Java Ocidental, Indonésia. Um questionário online foi distribuído para avaliar o estresse laboral e os dados foram analisados por meio de regressão logística ordinal.

**Resultados:**

Mais da metade dos enfermeiros relatou estresse laboral moderado. O estudo revelou que enfermeiros com mais de 35 anos tinham menor probabilidade de vivenciar estresse laboral (AOR: 0,173; IC95%: 0,038-0,782) e enfermeiros casados tinham maior probabilidade (AOR: 7,156; IC95%: 1,456-35,163). Enfermeiros com cargas de trabalho baixas e moderadas tinham menor probabilidade, (AOR: 0,003; IC95%: 0,000-0,051) e (AOR: 0,025; IC95%: 0,005-0,116), respectivamente.

**Conclusão:**

É essencial considerar a idade, estado civil e carga de trabalho para uma abordagem eficaz do estresse laboral entre enfermeiros.

## RESUMEN

**Objetivo:**

Analizar los factores contribuyentes del estrés laboral entre enfermeros en la pandemia de COVID-19.

**Métodos:**

Estudio transversal realizado con 101 enfermeros de dos hospitales de Java Occidental, Indonesia. Se distribuyó un cuestionario en línea para evaluar el estrés laboral y los datos se analizaron mediante regresión logística ordinal.

**Resultados:**

Más de la mitad de los enfermeros experimentaron un estrés laboral moderado. El estudio reveló que los enfermeros mayores de 35 años tenían una menor probabilidad de experimentar estrés laboral (AOR: 0.173; IC95%: 0.038-0.782) y los enfermeros casados tenían una mayor probabilidad (AOR: 7.156; IC95%: 1.456-35.163). Los enfermeros con cargas de trabajo bajas y moderadas tenían una probabilidad menor, (AOR: 0.003; IC95%: 0.000-0.051) y (AOR: 0.025; IC95%: 0.005-0.116), respectivamente.

**Conclusión:**

Es fundamental considerar la edad, el estado civil y la carga de trabajo para un abordaje eficaz del estrés laboral entre los enfermeros.

## INTRODUCTION

During the COVID-19 pandemic, nurses have faced significant challenges and heavy workloads as healthcare workers^(1)^. These conditions can lead to work-related stress among nurse practitioners working in hospitals, ultimately affecting the quantity and quality of services provided. Furthermore, work-related stress can have a significant impact on the health of individuals, communities, organizations, and even the national economy^(2,3)^. Research conducted in several countries, including Sweden, Thailand, and Indonesia, has revealed high prevalence rates of work-related stress, reaching 24.5 %, 26.2%, and 30%, respectively^(4,5,6)^. Consequently, addressing the issue of work-related stress among nurse practitioners in hospitals becomes a matter of serious concern.

Work-related stress among nursing refers to the physical, emotional, and psychological strain experienced by nurses as a result of their work environment and job demands^(7)^. Betty Neuman revealed her thoughts in the Neuman System model, stating that nurses naturally have a relationship with stress, response, and reconstitution factors in their lives^(8)^. High levels of work-related stress among nurses can compromise the quality of patient care^(9)^. Fatigue, decreased concentration, and emotional exhaustion can impact nurses’ ability to provide safe and effective care, potentially leading to medical errors and adverse patient outcomes^(10)^. Nurses play a vital role in promoting and protecting public health. Their involvement extends beyond direct patient care to areas such as health education, disease prevention, and community health promotion^(11)^. However, when nurses experience work-related stress, their capacity to engage in these public health activities may be diminished, thereby impacting population health outcomes^(7,12)^.

Previous studies have mentioned that demographic factors^(13)^, work environment, management and policies^(14)^, motivation, and the number of patients^(15)^ are contributed to work-related stress among practitioner nurses. This is concerning because high stress levels can lead to physical and mental health complications, cardiovascular disease, musculoskeletal disorders, anxiety, depression, and fatigue^(16)^. However, studies in Indonesia that specifically focus on exploring work-related stress among nurse practitioners during the COVID-19 pandemic are still limited^(17)^ and we found different results from previous studies^(18)^. Additionally, we pointed on age, marital status, and workload of nurses during the COVID-19 pandemic. Based on the research we conducted, it can provide knowledge and information suggesting that considering personal factors such as marital status can help reduce work-related stress, improve the quality of life for nurses, and have a positive impact on the quality of services provided.

## OBJECTIVE

The current study evaluates the contributing factors of work-related stress among nurse practitioners during the COVID-19 pandemic.

## METHODS

### Ethical consideration

The study adhered to ethical research principles, including self-determination, privacy and dignity, protection from discomfort and harm, and beneficence. It was approved by the Research Ethics Committee of Health Polytechnic of Tasikmalaya, Indonesia, with approval number KP-KEPK/0079/2022. Informed consent was obtained from all respondents participating in this study.

### Study design, period, and place

A cross-sectional study was conducted between June and July 2022. Nurse practitioners were recruited from two COVID-19 referral hospitals in West Java, Indonesia. This study used The Strengthening the Reporting of Observational Studies in Epidemiology (STROBE) statement guidelines to ensure the steps of the method and the quality^(19)^.

### Population or sample, inclusion, and exclusion criteria

The total population of nurses in West Java province was 23,592 people, based on the 2021 health profile data. The sample in the study consisted of 101 nurse practitioners who work in hospitals. The inclusion criteria were as follows: 1) a minimum working time of 1 year, 2) minimum vocational nursing education (Diploma III in nursing), and 3) experience in taking care of patients with COVID-19. Additionally, respondents were excluded if they were unable to complete the questionnaire, on sick leave, on vacation, or on time off.

### Data collection

The data collection process involved an online questionnaire distributed using the snowball technique through WhatsApp. Each participant was invited to participate and complete the questionnaire via the Google Forms application. The questionnaire aimed to gather data on factors associated with work-related stress among nurses during the COVID-19 pandemic. The data collection instrument consisted of several components, including individual characteristics such as age, gender, working duration, educational background, and marital status. Additionally, workload was assessed using a questionnaire comprised of 17 statements. The author adopted this nurse’s workload instrument from the previous study^(20)^. The questionnaire categorized workload into three levels: low, moderate, and high. A Likert scale was employed for this questionnaire. The score ranges for workload were as follows: low (17-33), moderate (34-50), and high (51-68). Work-related stress was determined using the Hamilton Anxiety Rating Scale (HARS), which typically ranges from 0 to 56. HARS categories were defined as low (<17), moderate(18-24), and high (25-30)^(21)^.

### Data analysis

The data analysis begins with screening the data obtained from the Google Form and exporting it to Microsoft Excel. Any missing data are excluded. The data is then analyzed using IBM SPSS Statistics version 26 for Windows. Descriptive analysis is performed to determine the data distribution, which is presented using numbers and percentages. Subsequently, a chi-square analysis is performed to examine the relationship between the independent variables and the dependent variables. After that, ordinal logistic regression is conducted to identify the risk and protective factors associated with work-related stress among nurse practitioners. The findings are reported using Adjusted Odds Ratio (AOR), 95% Confidence Interval (CI), and a p-value threshold of < 0.05.

## RESULTS

Out of the 101 nurse practitioners, we observed that 20 (19.8%) experienced low levels of work-related stress, 58 (57.4%) experienced moderate levels, and 23 (22.8%) experienced high levels. The demographic analysis revealed that the majority of respondents were over 35 years old, female, and had at least 5 years of nursing experience. In terms of education, most of them held a Bachelor of Nursing degree and were married. More than half of the respondents reported having a moderate workload. Furthermore, the chi-square analysis results indicated a significant relationship between all variables and work-related stress (*p* value < 0.001) (Table 1). Subsequently, an ordinal logistic regression analysis was conducted (Table 2).

**Table 1:** Characteristic respondents and chi-square analysis of work-related stress among practitioner nurses (N=101)

Characteristics	n (%)	Work-Relates Stress			X^2^
		Low (n=20) n (%)	Moderate (n=58) n (%)	High (n=23) n (%)	
Age					
≤ 35 years old	30 (29.7)	18 (60.0)	8 (26.7)	4 (13.3)	51.750***
>35 years old	71 (70.3)	2 (2.8)	50 (70.4)	19 (26.8)	
Gender					
Male	34 (33.7)	18 (52.9)	15 (44.1)	1 (2.9)	36.563***
Female	67 (66.3)	2 (3.0)	43 (64.2)	22 (32.8)	
Years of Service					
< 5 years	31 (30.7)	15 (48.4)	14 (45.2)	2 (6.5)	12.188***
≥ 5 years	70 (69.3)	5 (7.1)	44 (62.9)	21 (30.0)	
Educational Background					
Vocational	33 (32.7)	18 (54.4)	14 (42.4)	1 (3.0)	39.600***
Bachelor	68 (67.3)	2 (2.9)	44 (64.7)	22 (32.4)	
Marital Status					
Married	66 (65.3)	1 (1.5)	44 (66.7)	21 (31.8)	14.500***
Single	35 (34.7)	19 (54.3)	14 (40.0)	2 (5.7)	
Workload					
Low	16 (15.8)	10 (62.5)	6 (37.5)	0 (0.0)	
Moderate	55 (54.5)	8 (14.8)	46 (83.6)	1 (1.8)	12.500***
High	30 (29.7)	2 (6.7)	6 (20.0)	22 (73.3)	

*** *p value < 0.001.*

**Table 2:** Ordinal regression logistic analysis

Variables	P value	AOR	95% CI	
			Lower	Upper
Age	**0.023**	**0.173**	0.038	0.782
Gender	0.704	0.177	0.000	1358.315
Year of service	0.366	0.482	0.099	2.349
Education	0.800	0.313	0.000	2492.397
Marital status	**0.015**	**7.156**	1.456	35.163
Workload=mild	**< 0.001**	**0.003**	0.000	0.051
Workload=moderate	**< 0.001**	**0.025**	0.005	0.116

*AOR: Adjusted Odds Ration; CI: Confidence Interval*

Table 2 shows that age and workload are identified as protective factors, while marital status is identified as a risk factor for work-related stress among nurse practitioners. Nurses aged over 35 years have a 0.173 times lower likelihood of experiencing work-related stress compared to their younger counterparts (AOR: 0.173; 95% CI: 0.038-0.782). Furthermore, nurses with mild and moderate workloads exhibit a decreased likelihood of experiencing work-related stress compared to those with high workloads (AOR: 0.003; 95% CI: 0.000-0.051) and (AOR: 0.025; 95% CI: 0.005-0.116), respectively. Conversely, married nurses have a significantly higher likelihood of experiencing work-related stress, with 7,156 times greater odds compared to unmarried nurses (AOR: 7,156; 95% CI: 1,456-35,163). Gender, years of service, and education level variables do not demonstrate a significant relationship.

## DISCUSSION

From this study, it is known that age, marital status, and workload contribute significantly to work-related stress in nurse practitioners in hospitals during the COVID-19 pandemic. In this section, we discussed these factors and their relationship with work-related stress.

The COVID-19 pandemic has placed an unprecedented burden on healthcare professionals, particularly nurses, leading to heightened levels of work-related stress^(22,23)^. In our study, we discovered that more than half of the total respondents experienced a moderate level of work-related stress. The unique demands imposed by the pandemic, such as increased patient loads, resource shortages, and heightened exposure to the virus, have significantly contributed to the overwhelming stress experienced by nurses^(24)^. The constant fear of contracting the virus, witnessing the suffering and death of patients, and enduring long working hours have had a profound impact on their mental and emotional well-being^(25)^. The consequences of work-related stress among nurses during the pandemic are multifaceted and can adversely affect patient care, job satisfaction, and overall healthcare outcomes^(26)^. Recognizing the significant challenges faced by nurses and implementing effective strategies to mitigate work-related stress are crucial for promoting their well-being and ensuring optimal healthcare delivery during these demanding times.

This study found that nurses aged over 35 years were less likely to experience work-related stress during the COVID-19 pandemic. This suggests that with age, nurses may have developed better coping mechanisms and gained more experience in managing stress, allowing them to handle pandemic challenges more effectively. Aligns with the theory of age and coping, which posits that individuals tend to develop improved coping mechanisms and strategies as they age^(27)^. With years of experience in the nursing profession, older nurses may have acquired a repertoire of effective coping strategies, such as problem-solving skills through teamwork, health management, and patient care^(28,29,30)^. These coping mechanisms enable them to navigate the challenging and demanding nature of their work during the COVID-19 pandemic more effectively. Additionally, older nurses may have developed a greater sense of resilience, adapting to stressful situations, and maintaining a positive outlook^(31,32)^. In line with the previous studies highlighting the potential benefits of age and experience in mitigating stress levels in healthcare professionals^(33,34)^. However, further investigation is needed to better understand the underlying factors contributing to this phenomenon and to develop targeted interventions to support younger nurses who may be more susceptible to work-related stress.

We observed that married nurses had a higher likelihood of experiencing work-related stress. One plausible explanation could be the additional responsibilities and commitments that come with being married, such as managing household chores, taking care of children, and maintaining a work-life balance^(35,36)^. These added responsibilities may increase the overall stress levels experienced by married nurses, as they navigate the demands of both their personal and professional lives. Additionally, the COVID-19 pandemic has placed unprecedented strain on individuals and families, potentially exacerbating the stress experienced by married nurses^(37)^. Moreover, the pandemic has disrupted daily routines and increased the burden on individuals, including married nurses, who may have to juggle multiple roles and responsibilities simultaneously^(2)^. Additionally, the fear of potentially exposing family members to the virus due to their work as healthcare professionals can contribute to heightened stress levels among married nurses^(38)^. The importance of providing targeted support and resources to help married nurses manage their work-life balance, enhance their well-being, and reduce work-related stress during the COVID-19 pandemic should be implemented.

Nurses with a mild to moderate workload had a lower likelihood of experiencing work-related stress. This finding suggests that maintaining a balanced workload, neither overwhelming nor underwhelming, may contribute to reducing stress levels among nurses. A mild to moderate workload allows nurses to effectively manage their tasks and responsibilities without becoming over-whelmed^(39)^, ensuring they have adequate time and energy to cope with the challenges posed by the pandemic^(40)^. This finding emphasizes the importance of workload management strategies in healthcare settings, such as appropriate staffing levels, task delegation, and efficient scheduling, to help mitigate work-related stress among nurse practitioners during COVID-19 pandemic^(41,42)^. By ensuring workload remains within manageable limits, healthcare organizations can promote the well-being of their nursing staff and enhance the quality of patient care during the COVID-19 pandemic.

### Study limitations

The research methodology employed in this study has certain limitations that need to be considered. One limitation is its cross-sectional nature, which means that it is not possible to establish a causal relationship between the variables studied. The study design captures a snapshot of data at a specific point in time, preventing the researchers from making definitive conclusions about cause and effect. Another limitation pertains to the sample selection, which relied on convenience sampling. The sample consisted of nurses who had treated COVID-19 patients during the pandemic, excluding those who were on leave or not actively involved in patient care at the time. Additionally, it is worth noting that the sample was restricted to nurses from only two hospitals in the province of West Java. This limited scope may impact the generalizability of the findings to a broader population of nurses working in different regions or healthcare settings.

### Contributions to the field

Our findings suggest that hospital management should prioritize providing dedicated support to nurses who are involved in the care of COVID-19 patients. This support should focus on optimizing nurse workload by ensuring adequate staffing levels that align with the specific needs of the patients. Creating a supportive environment is also crucial, as it can help nurses navigate the challenges they face. Additionally, hospital management should assist nurses in prioritizing their various nursing tasks by establishing a scale of priorities and implementing standard operating procedures. Effective time management practices can also play a significant role in reducing work-related stress among nurses. It is important to pay special attention to younger nurses and provide them with appropriate coping mechanisms to mitigate their susceptibility to work-related stress. By implementing these strategies, hospital management can enhance the well-being of nurses, improve patient care, and foster a healthier work environment during the COVID-19 pandemic.

## CONCLUSION

The study revealed that age, marital status, and workloads were significant factors contributing to work-related stress among nurse practitioners during the COVID-19 pandemic. These findings provide valuable insights that can inform the development of policies aimed at preventing and addressing work-related stress among nurses in the hospital. By addressing these factors, the quality and quantity of nursing services can be enhanced, enabling nurses to deliver optimal care to patients, particularly those affected by COVID-19. Implementing strategies to alleviate work-related stress can positively impact the well-being of nurses and ultimately improve patient outcomes.
